# Chimeric Antigen Receptor-Engineered NK-92 Cells: An Off-the-Shelf Cellular Therapeutic for Targeted Elimination of Cancer Cells and Induction of Protective Antitumor Immunity

**DOI:** 10.3389/fimmu.2017.00533

**Published:** 2017-05-18

**Authors:** Congcong Zhang, Pranav Oberoi, Sarah Oelsner, Anja Waldmann, Aline Lindner, Torsten Tonn, Winfried S. Wels

**Affiliations:** ^1^Georg-Speyer-Haus, Institute for Tumor Biology and Experimental Therapy, Frankfurt am Main, Germany; ^2^German Cancer Consortium (DKTK), Partner Site Frankfurt/Mainz, Frankfurt am Main, Germany; ^3^German Cancer Research Center (DKFZ), Heidelberg, Germany; ^4^German Red Cross Blood Donation Service North-East, Institute for Transfusion Medicine, Dresden, Germany; ^5^Medical Faculty Carl Gustav Carus, TU Dresden, Dresden, Germany; ^6^German Cancer Consortium (DKTK), Partner Site Dresden, Dresden, Germany

**Keywords:** natural killer cells, NK-92, chimeric antigen receptor, adoptive cancer immunotherapy, leukemia, lymphoma, solid tumors

## Abstract

Significant progress has been made in recent years toward realizing the potential of natural killer (NK) cells for cancer immunotherapy. NK cells can respond rapidly to transformed and stressed cells and have the intrinsic potential to extravasate and reach their targets in almost all body tissues. In addition to donor-derived primary NK cells, also the established NK cell line NK-92 is being developed for adoptive immunotherapy, and general safety of infusion of irradiated NK-92 cells has been established in phase I clinical trials with clinical responses observed in some of the cancer patients treated. To enhance their therapeutic utility, NK-92 cells have been modified to express chimeric antigen receptors (CARs) composed of a tumor-specific single chain fragment variable antibody fragment fused *via* hinge and transmembrane regions to intracellular signaling moieties such as CD3ζ or composite signaling domains containing a costimulatory protein together with CD3ζ. CAR-mediated activation of NK cells then bypasses inhibitory signals and overcomes NK resistance of tumor cells. In contrast to primary NK cells, CAR-engineered NK-92 cell lines suitable for clinical development can be established from molecularly and functionally well-characterized single cell clones following good manufacturing practice-compliant procedures. In preclinical *in vitro* and *in vivo* models, potent antitumor activity of NK-92 variants targeted to differentiation antigens expressed by hematologic malignancies, and overexpressed or mutated self-antigens associated with solid tumors has been found, encouraging further development of CAR-engineered NK-92 cells. Importantly, in syngeneic mouse tumor models, induction of endogenous antitumor immunity after treatment with CAR-expressing NK-92 cells has been demonstrated, resulting in cures and long-lasting immunological memory protecting against tumor rechallenge at distant sites. Here, we summarize the current status and future prospects of CAR-engineered NK-92 cells as off-the-shelf cellular therapeutics, with special emphasis on ErbB2 (HER2)-specific NK-92 cells that are approaching clinical application.

## Introduction

Natural killer (NK) cells are specialized effectors of the innate immune system and central players in the defense against viral infections and cancer. Natural cytotoxicity of NK cells can be triggered rapidly upon appropriate stimulation and is regulated by a complex balance of signals from germ-line encoded activating and inhibitory cell surface receptors (1, 2). The antitumoral activity of NK cells has been well documented in mouse models (3, 4). In humans, a correlation between low peripheral blood NK-cell activity and an increased cancer risk was demonstrated (5), and numbers and phenotype of tumor-infiltrating NK cells likely influence the course of the disease (6–8). Mechanisms involved in tumor immune evasion can be diverse and include upregulation of the non-classical MHC molecules HLA-E and HLA-G that trigger inhibitory NK-cell receptors (9), selective loss of ligands for activating NK-cell receptors (10, 11), as well as shedding of soluble forms of MHC class I polypeptide-related sequence A/B (MICA/B) and B7-H6 (12–14). Furthermore, the tumor microenvironment plays a crucial role in preventing infiltration by NK and other immune cells and interfering with the activity of NK cells already present in the tumor (15, 16). Hypoxia as well as immunosuppressive factors such as transforming growth factor (TGF)-β, indoleamine 2,3-deoxygenase (IDO), prostaglandin E2, nitric oxide (NO), and reactive oxygen species (ROS), which are produced by regulatory immune cells like regulatory T (T_reg_) cells and myeloid-derived suppressor cells, by stromal cells like cancer-associated fibroblasts, and by tumor cells themselves can inhibit expression of activating NK-cell receptors, disrupt the interactions between NK and other immune cells, and avert the contact of NK cells with tumor cells (17).

To bypass deficiencies in endogenous NK-cell activity, current NK-cell therapies are typically based on adoptive transfer of *ex vivo*-expanded allogeneic NK cells derived from a suitable donor (18–20). While displaying graft-versus-leukemia (GvL) or graft-versus-tumor (GvT) activity, such donor-derived NK cells do not carry a high risk of inducing graft-versus-host-disease (GvHD) frequently associated with donor lymphocyte infusion (DLI) of allogeneic T cells (20). In addition, antibodies that block inhibitory NK-cell receptors such as killer cell immunoglobulin-like receptors (KIRs) and NKG2A/CD94, or link activating NK-cell receptors to tumor cell surface antigens are being investigated as activity enhancers for endogenous or adoptively transferred NK cells (21, 22). Sparked by the clinical success of chimeric antigen receptor (CAR)-engineered T cells in the treatment of B-cell malignancies, genetic modification of NK cells with CAR constructs is receiving increasing attention. CAR engagement in NK cells can override inhibitory signals deployed by tumor cells and directly trigger the effector cells’ intrinsic cytolytic effector functions as well as the release of pro-inflammatory cytokines (23, 24). Nevertheless, despite the close similarity of NK cells to T cells with respect to their cytotoxic mechanisms, the development of CAR-engineered NK cells for adoptive cancer immunotherapy is still in its early stages, owing mainly to the complexity of isolating, activating, expanding, and manufacturing large numbers of peripheral blood-derived NK cells, the lower efficiency of gene transfer when compared to T cells, and the limited *in vivo* proliferation and persistence in recipients. While efforts are being made to overcome these hurdles by improving *ex vivo* expansion of NK cells to allow multiple infusions (25), results from clinical trials with CAR NK cells are not yet available.

Continuously expanding NK cell lines provide an unlimited source of effector cells to investigate and improve concepts for genetic engineering of NK cells (23, 26–29) but also hold potential for development as standardized off-the-shelf therapeutics for adoptive cancer immunotherapy. Different human NK cell lines have been established, including NK-92, HANK-1, KHYG-1, NK-YS, NKG, YT, YTS, NKL, and NK3.3 (30). Among them, NK-92 cells (also termed “aNK” for activated NK) have been investigated most thoroughly and already been applied in a clinical setting (31, 32). NK-92 express many activating NK-cell receptors such as NKp30, NKp46, and NKG2D but lack most of the inhibitory KIRs, except for low levels of KIR2DL4 (33, 34). Other inhibitory receptors expressed by NK-92 are Ig-like transcript 2 (ILT-2) and NKG2A/CD94. This unique profile renders NK-92 cells highly cytotoxic against a broad spectrum of malignant cells of hematologic origin and other cancers (32). General safety of infusion of irradiated NK-92 cells has been established in phase I clinical trials in patients with advanced cancers (35, 36), and results from other phase I and phase II studies may soon become available (NCT00990717, NCT00900809, NCT02465957; https://clinicaltrials.gov).

As outlined in the following sections, the robust *ex vivo* expansion of NK-92 cells to high cell numbers, their exquisite safety profile, as well as the ease of genetic modification make this cell line an ideal platform for the development of CAR-engineered variants. Here, we provide an overview of the diverse approaches that have been taken to date to target NK-92 cells to various hematological malignancies and solid tumors, summarize preclinical *in vitro* and *in vivo* studies with special emphasis on ErbB2 (HER2)-specific CAR NK-92 cells (NK-92/5.28.z) that are ready to enter clinical trials, and discuss general advantages and challenges associated with the use of CAR NK-92 cells as an off-the-shelf cellular therapeutic.

## Advances from the CAR T Cell Field Enabling the Generation of Tumor-Specific NK Cells

Since introduction of the basic CAR design with a single chain fragment variable (scFv) antibody for target recognition fused to CD3ζ or FcεRIγ chains for signaling (first-generation CARs) by Eshhar and colleagues (37), many groups have contributed to further improve and develop this concept, facilitating the clinical success of CAR T cell therapy seen today (38, 39). The most significant CAR modification was thereby the inclusion of costimulatory protein domains derived from CD27, CD28, CD134 (OX40), CD137 (4-1BB), CD244 (2B4) or CD278 (ICOS) (second-generation CARs), or their combinations (third-generation CARs) in addition to CD3ζ to improve T-cell activation, proliferation, and persistence (40). Other advances enhancing CAR functionality and providing additional benefits with respect to stimulating innate immunity, improving safety, or alleviating tumor immune escape have been reviewed extensively by Fesnak et al. (41). They include for instance interleukin (IL)-12-armed T cells redirected for universal cytokine-mediated killing (TRUCKs) (42), universal CARs activated by modular antibody-based targeting molecules (43), and dual-targeting tandem CARs (TanCARs) (44, 45). In patients with lymphomas and leukemias of B-cell origin, remarkable efficacy was demonstrated and durable responses were achieved with both, T-cell products harboring CD19-specific second-generation CD28- or CD137-containing CARs. While in experimental models CD28-CD3ζ CARs led to stronger T-cell activation, CD137-CD3ζ CARs prolonged *in vivo* T-cell persistence and reduced exhaustion (46, 47).

Already early on, it was postulated for first-generation CARs that they would be functional in NK cells (37), which was formally demonstrated for a CAR-like CD4-CD3ζ fusion receptor in human NK3.3 cells (26). In the first report proposing CAR-engineered NK-92 cells as a continuously expanding off-the-shelf cell therapeutic, we also applied a first-generation CAR consisting of an ErbB2-specific scFv antibody fused to CD3ζ through a CD8α hinge region, which resulted in high and specific cytotoxicity of the genetically modified cells toward ErbB2-expressing breast cancer cells and other targets of solid tumor origins (23). Similar first-generation CAR designs were successfully used in subsequent studies with NK-92 cells targeting the B-cell differentiation antigens CD19 and CD20 (48–53), CD138 for recognition of multiple myeloma (54), and various surface antigens expressed by solid tumors including the disialoganglioside GD2, epithelial cell adhesion molecule (EpCAM), and a peptide epitope of the melanoma antigen gp100 in complex with HLA-A2 (55–59) (Table 1). In studies with CD19- and GD2-targeted primary human NK cells, inclusion of costimulatory CD137 or CD244 domains in the CAR in addition to CD3ζ enhanced both specific cytotoxicity and production of interferon (IFN)-γ and granulocyte-macrophage colony stimulating factor (GM-CSF) when compared to first-generation CARs (24, 60). This clearly demonstrates that at least primary NK cells benefit from CAR-induced costimulatory signals. In preclinical studies, also an ErbB2-specific CD28-CD3ζ CAR and a CD20-specific CD137-CD3ζ CAR were shown to be functional in donor-derived human NK cells, but no comparison with respective CD3ζ-only CARs was performed (61, 62). Clinical trials with CAR-engineered primary NK cells for the treatment of B-cell acute lymphoblastic leukemia (B-ALL) employ CD19-specific CD137-CD3ζ receptors (NCT00995137, NCT01974479; https://clinicaltrials.gov), but results from these trials are not yet available.

**Table 1:** **Preclinical studies with CAR NK-92 cells**.

Target	Antibody	Hinge	TM	Signaling	Gene transfer	Cancer type	*In vivo* model	Treatment	Reference
ErbB2 (HER2)	FRP5	mCD8α	mCD3ζ	mCD3ζ	Retrovirus	Breast ca. Ovarian ca. SCC	CD-1 nude	Local co-injection	Uherek et al. (23)
ErbB2 (HER2)	FRP5	mCD8α	mCD3ζ	mCD3ζ	Retrovirus	Breast ca.	BALB/c nude	Systemic	Daldrup-Link et al. (63), Meier et al. (64)
ErbB2 (HER2)	FRP5	mCD8α	mCD3ζ	mCD3ζ	Retrovirus	Brain metastasis	Athymic nude rats	Systemic (with FUS)	Alkins et al. (65), Alkins et al. (66)
ErbB2 (HER2)	4D5-8	hIgG2	hFcεRIγ	hFcεRIγ	Retrovirus	Breast ca.	NSG	Systemic	Clemenceau et al. (58)
ErbB2 (HER2)	FRP5	hCD8α	hCD3ζ hCD28 hCD137	hCD3ζ hCD28-CD3ζ hCD137-CD3ζ	Lentivirus	Breast ca. Ovarian ca. Melanoma RCC	NSG	Systemic	Schönfeld et al. (67)
ErbB2 (HER2)	n.s.	hCD8α	hCD28	hCD28-CD3ζ	Electroporation	Breast ca.	BALB/c nude	Systemic	Liu et al. (68)
ErbB2 (HER2)	FRP5	hCD8α	hCD28	hCD28-CD3ζ	Lentivirus	GBM	NSG C57BL/6	Local	Zhang et al. (69)
Epidermal growth factor receptor (EGFR)	528	n.s.	n.s.	hCD28-CD3ζ	Lentivirus	Brain metastasis	NSG	Local (combined with HSV-1)	Chen et al. (70)
EGFR	R-1	hCD8α	hCD28	hCD28-CD3ζ	Lentivirus	GBM	NSG	Local	Genßler et al. (71)
EGFR/EGFRvIII	528	n.s.	hCD28	hCD28-CD3ζ	Lentivirus	GBM	NSG	Local	Han et al. (72)
EGFR/EGFRvIII	225	hCD8α	hCD28	hCD28-CD3ζ	Lentivirus	GBM	NSG	Local	Genßler et al. (71)
EGFRvIII	MR1-1	hCD8α	hCD28	hCD28-CD3ζ	Lentivirus	GBM	NSG	Local	Genßler et al. (71)
GD2	ch14.18	mCD8α	mCD3ζ	mCD3ζ	Retrovirus	NB Breast ca. Melanoma	NSG	Local	Esser et al. (56), Seidel et al. (59)
Epithelial cell adhesion molecule (EpCAM)	MOC31	mCD8α	mCD3ζ	mCD3ζ	Retrovirus	Prostate ca.	Athymic nude rats	Systemic	Tavri et al. (55), Meier et al. (73)
EpCAM	MOC31	hCD8α	hCD28	hCD28-CD3ζ	Lentivirus	Breast ca.	–	–	Sahm et al. (28)
CD19	FMC63	mCD8α	mCD3ζ	mCD3ζ	Retrovirus	B-ALL	–	–	Romanski et al. (48), Romanski et al. (53)
CD19	FMC63	mCD8α	mCD3ζ	mCD3ζ	mRNA transfection	B-ALL CLL	–	–	Boissel et al. (50)
CD19	FMC63	mCD8α	mCD3ζ	mCD3ζ	mRNA transfection Lentivirus	B-ALL	CLL	Burkitt’s lymphoma	–	–	Boissel et al. (51)
CD19	FMC63	mCD8α	mCD3ζ	mCD3ζ	Lentivirus	B-ALL CLL	NOD/SCID NSG	Local Systemic	Boissel et al. (52)
CD19	FMC63	hCD8α	hCD3ζ hCD28 hCD137	hCD3ζ hCD28-CD3ζ hCD137-CD3ζ	Lentivirus	B-ALL Burkitt’s lymphoma	NSG	Systemic	Oelsner et al. (74)
CD20	Leu-16	mCD8α	mCD3ζ	mCD3ζ	Retrovirus	B-ALL CLL Burkitt’s lymphoma	NSG	Local co-injection	Müller et al. (49)
CD20	Leu-16	mCD8α	mCD3ζ	mCD3ζ	mRNA transfection	B-ALL	CLL	Burkitt’s lymphoma	–	–	Boissel et al. (51)
Lentivirus
CD20	Leu-16	mCD8α	mCD3ζ	mCD3ζ	Lentivirus	B-ALL	NOD/SCID	Local	Boissel et al. (52)
CLL	NSG	Systemic
CD3	n.s.	hCD8α	hCD8α	hCD28-CD137-CD3ζ	Lentivirus	PTCL	NSG	Systemic	Chen et al. (75)
T-ALL
CD5	n.s.	hCD8α	hCD8α	hCD28-CD137-CD3ζ	Lentivirus	PTCL T-ALL CLL Burkitt’s lymphomaSézary syndrome	NSG	Systemic	Chen et al. (76)
CD138	4B3	hCD8α	hCD3ζ	hCD3ζ	Lentivirus	MM	NOD/SCID	Systemic	Jiang et al. (54)
CS1	Luc90	n.s.	n.s.	hCD28-CD3ζ	Lentivirus	MM	NSG	Systemic	Chu et al. (77)
EBNA3C peptide	EBNA Clone 315	hCD8α	hCD8α	hCD137-CD3ζ	Retrovirus	BLCL	–	–	Tassev et al. (78)
gp100_209–217_ peptide	GPA7	n.s.	HLA-A2	hCD3ζ	Electroporation	Melanoma	NOD/SCID	Systemic	Zhang et al. (57)
WT1_126_ peptide	Q2L	hCD8α	hCD8α	hCD137-CD3ζ	Retrovirus	B-ALL AMoL NB	–	–	Zhao et al. (79)

*TM, transmembrane domain; n.s., not specified; m, murine; h, human; SCC, squamous cell carcinoma; RCC, renal cell carcinoma; GBM, glioblastoma; NB, neuroblastoma; B-ALL, B-cell acute lymphoblastic leukemia; CLL, chronic lymphocytic leukemia; PTCL, peripheral T-cell lymphoma; T-ALL, T-cell acute lymphoblastic leukemia; MM, multiple myeloma; BLCL, Epstein–Barr virus (EBV)-transformed lymphoblastoid B cell line; AMoL, acute monocytic leukemia; FUS, MRI-guided focused ultrasound*.

## Influence of the CAR Design on Functionality of Retargeted NK-92 Cells

In the presence of IL-2, NK-92 cells persistently exhibit a phenotype similar to activated NK cells (33). Hence, CAR-engineered NK-92 variants may be less dependent on costimulation than T cells and primary NK cells (80). Nevertheless, second-generation CARs employing a composite CD28-CD3ζ signaling domain have been shown to be functional in NK-92 cells targeting EpCAM and ErbB2 on breast cancer cells (28, 67, 68), epidermal growth factor receptor (EGFR) on glioblastoma cells and breast cancer brain metastases (70–72), EGFRvIII, a glioblastoma-specific mutant form of EGFR arising from an in-frame deletion of exons 2-7 of the receptor (71, 72), CD19 on B-cell malignancies (74), CS1 on multiple myeloma cells (77), and CD33 on acute myeloid leukemia cells (81). Likewise, second-generation CARs harboring CD137-CD3ζ domains and targeting ErbB2 (67), CD19 (74), or peptide epitopes of Epstein–Barr virus (EBV) latent protein EBNA3C, and Wilms tumor protein in complex with HLA-A2 (78, 79) have been used successfully with NK-92 cells as well as third generation CD28-CD137-CD3ζ CARs that recognize CD3 or CD5 for elimination of malignant T cells (75, 76) (Table 1).

**Figure 1:**
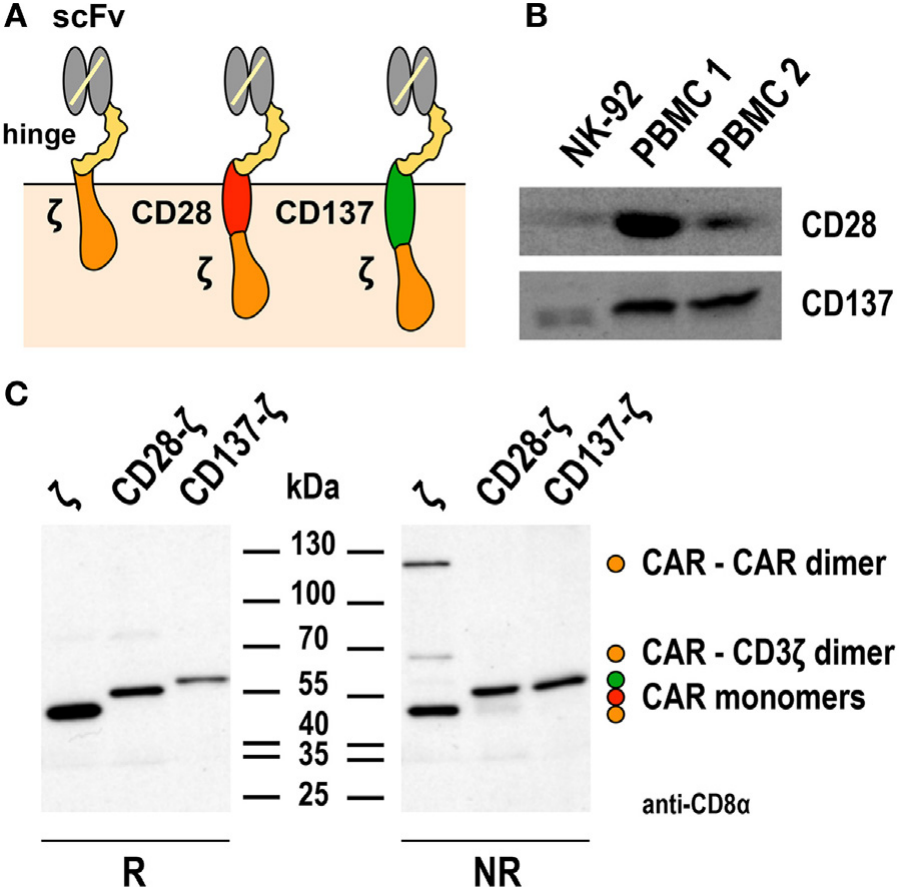
**Expression of first- and second-generation chimeric antigen receptors (CARs) in NK-92 cells**. **(A)** Schematic representation of first- and second-generation CARs for expression in NK-92 cells that consist of an extracellular single chain fragment variable (scFv) antibody domain for target recognition fused *via* a hinge region derived from CD8α (hinge) to transmembrane and intracellular domains of CD3ζ (left), transmembrane and intracellular domains of CD28, and the intracellular domain of CD3ζ (middle), or transmembrane and intracellular domains of CD137 (4-1BB) and the intracellular domain of CD3ζ (right). **(B)** To assess endogenous expression of CD28 and CD137, lysates of NK-92 cells were subjected to SDS-PAGE and subsequent immunoblotting with CD28- and CD137-specific antibodies as indicated. Lysates of peripheral blood mononuclear cells (PBMCs) from healthy donors were included for comparison. **(C)** For analysis of CAR expression, lysates of NK-92 cells transduced with lentiviral vectors that encode CD19-specific CARs containing CD3ζ, composite CD28-CD3ζ, or CD137-CD3ζ signaling domains as represented in **(A)** were subjected to SDS-PAGE under reducing (R, left panel) or non-reducing conditions (NR, right panel) and subsequent immunoblotting with CD8α-specific antibody, which detects the hinge domain. The positions of CAR monomers, homodimers of the CD3ζ CAR, and heterodimers of the CD3ζ CAR with endogenous CD3ζ are indicated. Data in panel **(C)** are from Oelsner et al. (74).

Only two reports compared the functionality of NK-92 cells harboring CD3ζ-based first-generation or CD28-CD3ζ- and CD137-CD3ζ-based second-generation CARs directly (67, 74), using a general CAR design as depicted in Figure 1A. NK-92 cells express high levels of CD3ζ and moderate levels of CD28 and CD137 (23, 34, 67) (Figure 1B), suggesting that the CARs could readily link to respective endogenous signaling pathways. Indeed, while differences were relatively small, ErbB2-targeted NK-92 cells expressing CD28-CD3ζ and CD137-CD3ζ CARs displayed more pronounced cytotoxicity in short-term assays when compared to a corresponding CD3ζ-only CAR (67). Conversely, CD19-targeted NK-92 cells harboring a CD137-CD3ζ CAR were much less effective in cell killing than cells expressing a CD3ζ-only or a CD28-CD3ζ CAR containing the same cell targeting domain (74). With respect to cytokine production, highest amounts of IFN-γ were found in cultures of CD19-specific NK-92 expressing a CD28-CD3ζ CAR, while less pronounced levels were secreted upon CAR activation by cells harboring a CD3ζ-only CAR, and only marginally enhanced levels by cells carrying the CD137-CD3ζ CAR.

The first-generation CARs included in these studies for comparison utilized the endogenous transmembrane domain of CD3ζ. This allowed formation of both, disulfide-linked CAR homodimers, and heterodimers of the CAR with endogenous CD3ζ of NK-92 cells (67, 74). Such preformed receptor complexes may get activated more rapidly and by lower target antigen densities than the CARs with CD28-CD3ζ and CD137-CD3ζ domains, which contained the transmembrane domains of CD28 and CD137 and did not form covalent dimers as assessed by SDS-PAGE and immunoblot analysis (74) (Figure 1C). Of note, while this was not the case for the CD19-specific CD137-CD3ζ CAR tested in NK-92 cells, a different CD19-specific CD137-CD3ζ CAR that contained the transmembrane domain of CD8α and formed covalent CAR homodimers showed enhanced activity in comparison to a respective CD3ζ-only CAR in primary NK cells (24). Also sterical effects such as distance of the target epitope to the cell surface and CAR accessibility can play a role in determining the activation threshold of individual CARs (82, 83). This may explain why in NK-92 cells otherwise identical ErbB2- and CD19-targeted CD137-CD3ζ CARs in one case led to higher and in the other case to lower-specific cell killing when compared to the respective CD3ζ-only CAR (67, 74). Hence, while the data available so far suggest that inclusion of a costimulatory protein domain in the CAR can be beneficial at least for particular functions of NK-92 cells, continuing research efforts are needed to clarify whether cytotoxicity, cytokine production, and resistance to immunosuppressive mechanisms can be improved with a single, generalized CAR design. Possibly, the most optimal CAR composition has to be determined experimentally in each case, taking into consideration CAR-binding affinity, location of the binding epitope within the target antigen, length of hinge region, and nature of the transmembrane domain (84).

## Continuous Expansion of CAR NK-92 Cells

Isolation and *ex vivo* expansion of peripheral blood-derived NK cells for therapeutic applications can be demanding, time-consuming, and costly (85). Since KIR-mismatched allogeneic NK cells are superior to autologous cells, a suitable donor needs to be identified to allow for efficient GvL or GvT activity (18–20). Moreover, owing to the intricate heterogeneity of human NK cells with respect to cytotoxic and regulatory activity, NK-cell licensing, unlicensing, and memory, selecting the most appropriate NK subpopulations for cancer therapy is difficult (86, 87). Sufficient numbers of NK cells are critical for a better clinical outcome, which is complicated by the limited *ex vivo* expansion potential of NK cells that remains a challenge despite the development of genetically engineered feeder cells supporting NK-cell growth and improved protocols for cytokine stimulation (25, 88, 89). These issues are also relevant for the development of CAR-engineered primary NK cells, which may explain the slow progress in this field with respect to CAR T cells.

Chimeric antigen receptor-engineered NK-92 could offer a valid and cost-effective alternative to primary CAR NK or T cells, in particular, in cases, where a suitable donor is not available or the sophisticated infrastructure needed for cell isolation, expansion, and genetic modification is missing. Methodology for continuous good manufacturing practice (GMP)-compliant expansion from an established master cell bank has been validated in the framework of early phase clinical trials with unmodified NK-92 cells and can easily be adapted for large-scale production in centralized facilities (32, 90). This advantage may readily be extended to CAR-engineered NK-92 variants. In contrast to CAR approaches based on autologous or donor-derived primary cells, genetic modification of NK-92 cells is thereby not performed in a patient-individual setting under tight time constraints. Instead, a molecularly and functionally well-characterized cell product can be established for a particular target specificity independent from the time point of therapeutic application. The resulting cells are stable with respect to CAR expression and functionality during extended expansion, as recently demonstrated for ErbB2-specific NK-92/5.28.z cells (also termed “HER2.taNK” for HER2-specific target-activated NK), a single-cell clone derived under GMP-compliant conditions that is intended for clinical use (67).

As NK cells, NK-92 as well as their CAR-expressing derivatives are dependent on exogenous IL-2 for growth and maintenance of their activated phenotype (23, 33). To ease cell expansion, different groups have engineered NK-92 by retroviral transduction or particle-mediated non-viral gene transfer to ectopically produce IL-2, leading to IL-2 secretion and growth of the cells in the absence of IL-2 supplementation (91, 92). Similarly, IL-15 and stem cell factor (SCF) have been ectopically expressed in NK-92 using plasmid DNA transfection (93, 94). While the resulting cells proliferated in medium with lower IL-2 concentrations than parental NK-92, in contrast to the IL-2-engineered variants, they were not completely independent from exogenous cytokines. In humans, high concentrations of IL-2 are associated with severe toxicity. Furthermore, in contrast to IL-15, IL-2 preferentially enhances the activity of T_reg_ cells, which is not desired in the context of cancer immunotherapy (95). Hence, a modified version of IL-2 was developed for expression in NK-92, which carries a C-terminal KDEL endoplasmic reticulum retention signal. This still allowed activation of IL-2 receptor complexes in the secretory pathway of the producer cells but limited release of IL-2 and availability to bystander cells (96, 97). A similar effect was achieved by expression of unmodified IL-15 in NK-92 using a lentiviral vector, which supported growth in the absence of exogenous IL-2 but also restricted cytokine activity to the producer cells. When IL-15 was coexpressed with an EpCAM-specific CAR from a bicistronic lentiviral vector, transduced cells could be enriched merely by IL-2 withdrawal, with the selected CAR NK-92 cells displaying high and specific cytotoxicity in the absence of exogenous cytokines (28).

## CAR-Engineered NK-92 Cells Exhibit Antibody-Dependent Cell-Mediated Cytotoxicity (ADCC)-Like Activity and Serial Killing

Antibody-dependent cell-mediated cytotoxicity of NK cells is triggered by FcγRIIIa (CD16), which associates with CD3ζ and FcεRIγ that are linked to overlapping as well as distinct intracellular signaling pathways (98, 99). NK-92, which is phenotypically CD16-negative, readily mediates ADCC in the presence of a suitable IgG antibody when engineered to express FcγRIIIa (27, 97, 100). This has sparked efforts to clinically develop genetically modified NK-92 cells that harbor the high affinity V158 variant of CD16 (termed haNK) in combination with antibodies of IgG1 isotype (32, 97). Initial safety assessment of such cells in cancer patients is expected to begin soon (NCT03027128; https://clinicaltrials.gov). Interestingly, side by side comparison of NK-92 cells carrying a CD20-specific first-generation CAR with a CD3ζ domain showed more pronounced killing of otherwise NK-resistant primary CLL cells than CD16-engineered NK-92 applied together with rituximab (52). Similarly, NK-92 cells harboring an EBV EBNA3C-specific CAR lysed peptide-pulsed B-cell lymphoblastic cells more efficiently than CD16-engineered NK-92 in the presence of an anti-EBNA3C-Fc fusion protein (78) and NK-92 cells expressing a trastuzumab-based ErbB2-specific CAR with an FcεRIγ signaling domain displayed more enhanced cytotoxicity against breast carcinoma cells than NK-92 harboring a CD16-FcεRIγ hybrid receptor in combination with trastuzumab antibody (58).

**Figure 2:**
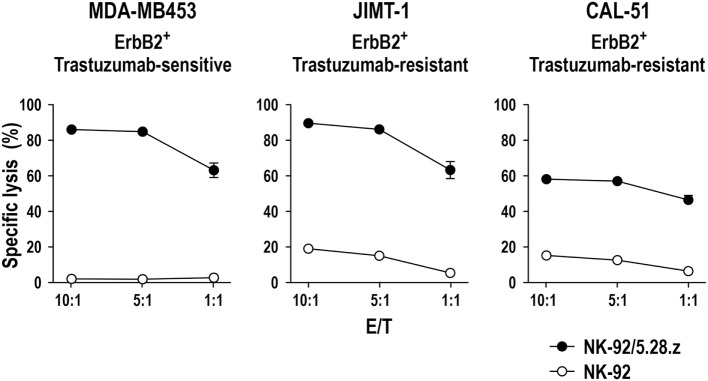
**Activity of NK-92/5.28.z against ErbB2-expressing breast carcinoma cells**. Cytotoxicity of CAR-engineered ErbB2-specific NK-92/5.28.z cells (filled circles) against ErbB2-overexpressing and trastuzumab-sensitive MDA-MB453 (left), or ErbB2-overexpressing and trastuzumab-resistant JIMT-1 (middle) and CAL-51 (right) breast carcinoma cells was investigated in flow cytometry-based cytotoxicity assays after coincubation of NK cells and tumor cells at different effector to target ratios (E/T) for 2 h. Parental NK-92 cells were included for comparison (open circles). Mean values ± SEM are shown; *n* = 3.

Successful triggering of ADCC through CD16 requires its non-covalent interaction with the Fc portion of an antibody that is simultaneously bound to its antigen on the surface of a neighboring target cell, as well as association with intracellular CD3ζ and FcεRIγ. Direct linkage of extracellular target recognition and intracellular signaling functions in one molecule as implemented in a CAR can bypass such complex stoichiometry and intermolecular interactions, likely accelerating kinetics of NK-cell activation. CAR signal strength is further enhanced by integrating CD3ζ, which in monomeric form contributes three immunoreceptor tyrosine-based activation motifs (ITAMs) that are crucial for downstream signaling, while an FcεRIγ monomer only provides one ITAM sequence (101). Accordingly, specific target-cell recognition by CAR NK-92 results in immediate and effective ADCC-like activity, characterized by orientation of cytotoxic granules toward the immunological synapse, release of high levels of perforin and granzyme B, and rapid induction of target-cell apoptosis as demonstrated for various tumor-associated antigens (23, 49, 67, 71, 74). Live cell imaging and cytotoxicity experiments at effector to target ratios below 1:1 showed that one CAR-engineered NK-92 cell can thereby kill multiple targets within a few hours (49, 67, 74). This includes tumor cells exhibiting only moderately enhanced expression of the chosen target antigen, as demonstrated for established and tumor-initiating primary glioblastoma cells exposed to ErbB2-specific NK-92/5.28.z cells (69). NK-92/5.28.z cells also killed trastuzumab-sensitive and trastuzumab-resistant ErbB2-positive breast carcinoma cells to a similar extent (Figure 2), attesting to the different mode of action of the retargeted NK cells and suggesting their application in a disease setting with existing resistance to other targeted therapies.

## *In Vivo* Antitumor Activity of CAR NK-92 Cells

Initial studies performed with ErbB2-, CD20-, and GD2-targeted CAR NK-92 cells showed that these cells retain specific cytotoxicity in simplified *in vivo* models in immunocompromised nude and NOD-SCID IL2R γ^null^ (NSG) mice, where effector cells were either subcutaneously coinjected together with tumor cells, or established subcutaneous tumors treated by peritumoral NK-cell injection. This resulted in delayed tumor onset and extended survival when compared to animals receiving parental NK-92 cells (23, 49, 59). Similar intratumoral treatment may be an option for cancer indications such as glioblastoma and brain metastasis, where disease is locally restricted. This has been investigated with NK-92 cells-expressing second-generation CARs targeting ErbB2, EGFR, or mutant EGFRvIII, which are expressed by a large proportion of human glioblastomas. In orthotopic xenograft models in NSG mice, repeated stereotactic injection of ErbB2-specific NK-92/5.28.z cells into the tumor area effectively inhibited tumor progression and resulted in a marked extension of survival, while parental NK-92 cells were ineffective (69). Similar effects were seen upon local application of NK-92 cells equipped with CARs that recognize EGFR, mutant EGFRvIII, or both antigens against orthotopic EGFR- and/or EGFRvIII-positive glioblastoma xenografts or breast cancer brain metastases growing in NSG mice (70–72). In contrast to EGFR- or EGFRvIII-targeted monospecific NK-92 variants, dual targeting of EGFR and EGFRvIII with a cetuximab-based CAR recognizing a common epitope of the receptors, thereby circumvented immune escape in mixed tumors that similar to the clinical situation, consisted of EGFR-positive and EGFR/EGFRvIII-double positive glioblastoma cells (71).

For broad applicability in metastatic and disseminated disease, CAR effector cells must cross tissue barriers and reach distant tumor sites to be effective. Magnetic resonance imaging, bioluminescence imaging, and positron emission tomography experiments as well as direct analysis of tumor infiltration revealed rapid and specific accumulation of intravenously injected NK-92 carrying first- and second-generation ErbB2-specific or EpCAM-specific CARs in orthotopic breast and subcutaneous prostate carcinoma xenografts in rodents (55, 63, 64, 67, 73), while parental NK-92 cells showed no tumor homing and were mainly localized to spleen and liver (55). Focused ultrasound has been demonstrated to allow systemically applied CAR NK-92 cells to cross the blood–brain barrier and reach breast cancer brain metastases in a xenograft model in immunocompromised rats (65, 66). Specific antitumor activity of intravenously applied CAR NK-92 cells has also been found in a model of locally growing breast carcinoma (68), in an experimental renal cell carcinoma metastasis model (67), and models of disseminated leukemia, lymphoma, and multiple myeloma (52, 74–77), underscoring the potential of CAR-engineered NK-92 cells for the treatment of a large variety of different cancers.

## NK Cells: A Bridge Between Innate and Adaptive Antitumor Immunity

**Figure 3:**
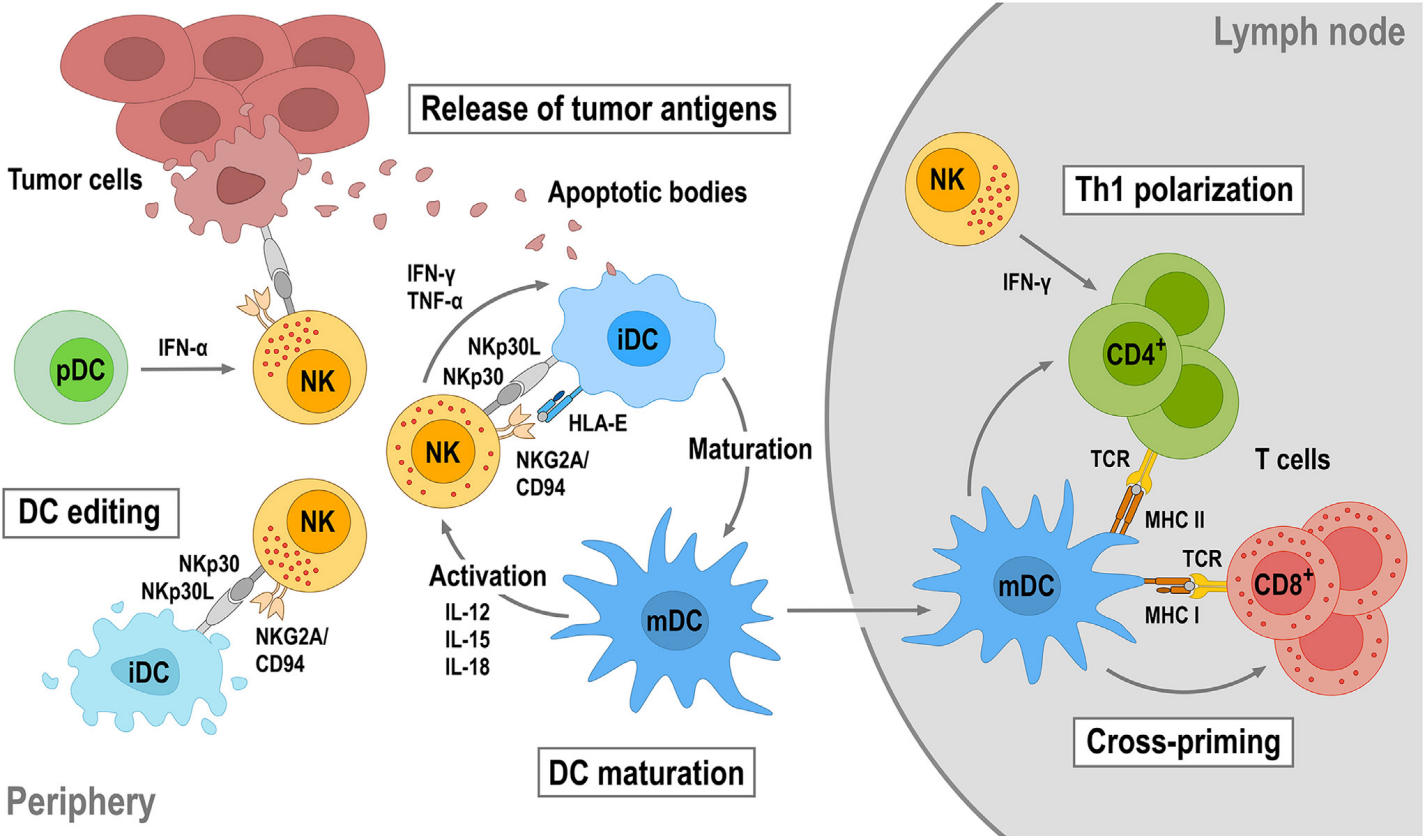
**Reciprocal natural killer (NK)—dendritic cell (DC) cross talk**. Upon activation by target tumor cells or cytokines, NK cells produce IFN-γ and tumor necrosis factor (TNF)-α that can promote DC maturation. DC maturation is also strongly dependent on the engagement of activating receptors on NK cells such as NKp30 and NKG2D. Mature DCs (mDCs) will in turn produce interleukin (IL)-12, IL-15, and IL-18, which enhance cytotoxicity and IFN-γ secretion of NK cells. NK cells can also distinguish immature (iDC) and mDCs through activating NKp30 and inhibitory killer cell immunoglobulin-like receptors and NKG2A/CD94 and eliminate immature DCs (iDCs), thereby maintaining the quality of the mDC population (DC editing). NK-cell cytotoxicity can be further augmented by IFN-α secreted by plasmacytoid DCs (pDCs). NK-induced tumor cell lysis provides antigens, which can be taken up by DCs for antigen presentation. Once maturated, antigen-loaded mDCs will migrate into tumor-draining lymph nodes, cross-present tumor antigens to naïve T cells, and induce their differentiation toward tumor-specific CD8^+^ cytotoxic T cells and CD4^+^ T helper 1 (Th1) cells.

Natural killer cells do not only play a critical role in antitumor immunity by directly eliminating malignant cells, but also by regulating tumor-specific adaptive immune responses through cross talk with other immune cells. In particular, the interaction between NK cells and dendritic cells (DCs) is important in this context (Figure 3). On the one hand, DCs enhance the direct antitumor activity of NK cells (102). On the other hand, NK cells regulate DC maturation, thereby determining the effectiveness of subsequent DC-mediated T-cell activation (103, 104). Once activated by target cells or soluble factors, NK cells secrete high amounts of IFN-γ and tumor necrosis factor (TNF)-α, which synergistically contribute to the maturation of immature DCs (iDCs). This leads to enhanced expression of costimulatory molecules such as CD80, CD83, and CD86 by the DCs and favors Th1 polarization during subsequent DC-mediated T-cell activation (105–107). Mature DCs (mDCs) release IL-12, IL-15, and IL-18, which in turn enhance IFN-γ expression by NK cells and NK-cell cytotoxicity against virus-infected and tumor cells (103, 108). Likewise, cytotoxicity of NK cells can be boosted by type I interferons such as IFN-α secreted by plasmacytoid DCs (pDCs) (109).

Dendritic cell maturation and reciprocal NK-cell activation are also strongly dependent on the engagement of activating receptors like NKp30, NKG2D, and NKp46 on NK cells (107, 110–113). Concurrent with inducing DC maturation, NK cells control the quality of the mDC population by killing iDCs (DC editing), which can otherwise induce immune tolerance through T-cell depletion or T_reg_ expansion. Discrimination and lysis of iDCs by NK cells is mainly regulated by activating signals through NKp30 and inhibitory signals through KIRs and the NKG2A/CD94 complex (107, 114). Accordingly, inhibition of NKp30 signaling or reduced NKp30 expression results in impaired NK-mediated killing of iDCs (110, 115, 116). Both mDCs and iDCs express NKp30 ligands. However, higher amounts of HLA class I and HLA-E molecules expressed by mDCs protect them from lysis by NK cells, whereas lower HLA class I and HLA-E expression makes iDCs vulnerable (114, 117). Importantly, by direct lysis of malignant cells, NK cells also provide tumor antigens for uptake and processing by DCs, which upon maturation and migration into a tumor-draining lymph node can cross-present such antigens to T cells, thereby inducing Th1 polarization of CD4^+^ T cells and differentiation of CD8^+^ T cells into tumor-specific cytotoxic T-lymphocytes (CTLs). NK cells can also migrate into tumor-draining lymph nodes and provide an early source of IFN-γ for Th1 polarization (118).

## CAR-Engineered NK-92 Cells Overcome Immunosuppressive Mechanisms and Enhance Adaptive Antitumor Immunity

As discussed above, efficient NK-cell activation is a prerequisite for productive NK–DC interaction. However, in cancer patients, NK-cell abnormalities are frequently found, including reduced NK-cell numbers, impaired cytotoxicity, and inefficient tumor infiltration (119). Especially in solid tumors, NK-cell activity is negatively affected by immunosuppressive factors in the tumor microenvironment (17, 120). High levels of TGF-β, IDO, and PGE2, as well as hypoxic conditions strongly inhibit the ability of NK cells to upregulate cytokine production and expression of activating cell surface receptors, while decreasing expression of ligands for activating NK-cell receptors by tumor cells. Under these conditions, tumor cells can also upregulate the non-classical MHC class I molecule HLA-G, a ligand for the NK-cell inhibitory receptors KIR2DL4 and ILT-2 (17, 121). Hence, therapeutic approaches that restore diminished NK-cell function may not only enhance direct NK-mediated tumor cell lysis but also improve clinical outcome by reinforcing DC activity and induction of adaptive antitumor immune responses.

Killing of cancer cells by CAR-engineered NK-92 is largely independent from the activation of endogenously expressed activating NK receptors and the presence of their ligands on target cells but mainly mediated by CAR-activation through binding to a cognate tumor-associated surface antigen (23, 49, 56, 67, 69, 71, 74). As recently demonstrated for ErbB2-specific NK-92/5.28.z carrying a CD28-CD3ζ CAR, such cells retain efficient CAR-mediated cell killing even under hypoxic conditions and in the presence of TGF-β concentrations exceeding the elevated TGF-β levels found in the plasma of cancer patients (69, 122). Furthermore, target tumor cells ectopically overexpressing human HLA-G were unable to block specific cell killing by CAR-engineered NK-92 (Zhang et al., unpublished data), although NK-92 cells express the immunoregulatory receptors KIR2DL4 and ILT-2, which are activated by HLA-G (34, 123). These findings show that activated CAR NK-92 cells can maintain their cytotoxic potential in an immunosuppressive environment similar to the one found within a solid tumor. In addition, NK-92 readily express activating NK receptors such as NKp30 and NKG2D while most of the inhibitory KIRs are absent (34), which may make CAR NK-92 cells particularly effective in aiding DC maturation and editing, and enhancing DC-mediated cross-priming of tumor-specific T cells and induction of adaptive antitumor immunity.

**Figure 4:**
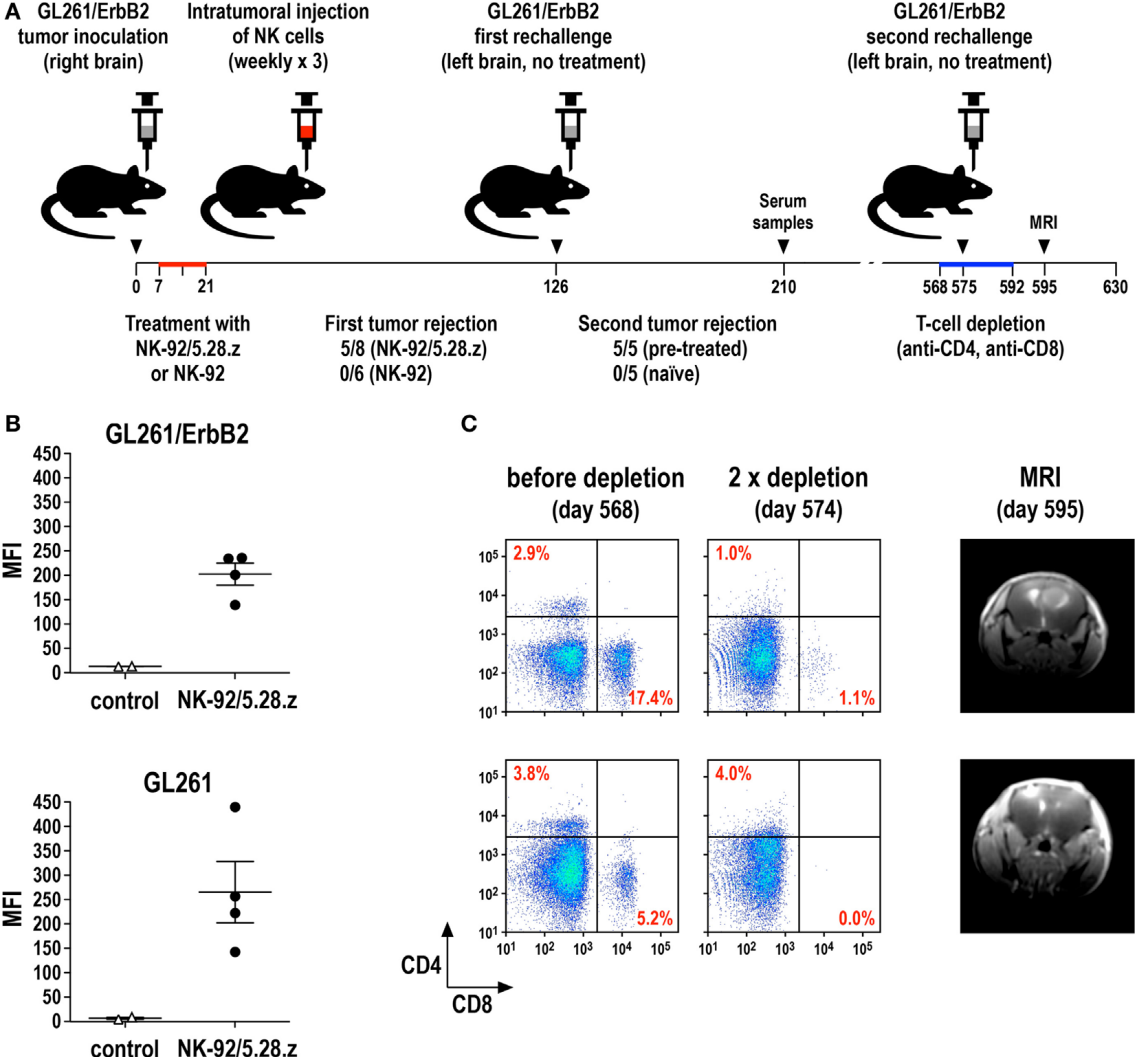
**Induction of protective antitumor immunity by CAR-engineered NK-92 cells**. **(A)** Murine GL261/ErbB2 glioblastoma cells (5 × 10^3^) stably expressing human ErbB2 were stereotactically injected into the right striatum of syngeneic C57BL/6 mice. Seven days later, animals were treated once per week for 3 weeks by intratumoral injection of 2 × 10^6^ parental NK-92 (*n* = 6) or NK-92/5.28.z cells (*n* = 8), which express an ErbB2-specific CAR with CD28 and CD3ζ signaling domains. Animals that were cured upon NK-92/5.28.z treatment (*n* = 5) were rechallenged at day 126 by injection of 5 × 10^3^ GL261/ErbB2 cells into the left brain hemisphere without further NK-cell therapy and symptom-free survival was followed. Naïve C57BL/6 mice injected into the brain with GL261/ErbB2 cells at day 126 served as a control (*n* = 5) (69). **(B)** Induction of IgG serum antibodies against glioblastoma cells in NK-92/5.28.z-treated animals (*n* = 4) from the experiment summarized in **(A)** was investigated by flow cytometry with GL261/ErbB2 (upper panel) and ErbB2-negative parental GL261 cells (lower panel) using sera collected at day 210. Sera from naïve C57BL/6 mice (*n* = 2) served as controls. MFI, mean fluorescence intensity (geometric mean). Data for GL261/ErbB2 cells are from Zhang et al. (69). **(C)** T cells in the NK-92/5.28.z-pretreated and rechallenged mice were depleted by intravenous injection of CD4- and CD8-specific antibodies at days 568, 573, 578, 582, 587, and 592 (left and middle panels). At day 575, these animals were rechallenged a second time with 5 × 10^3^ GL261/ErbB2 cells injected into the left brain hemisphere without further NK-cell therapy. Tumor development was assessed by MRI at day 595 (right panels).

We recently investigated this possibility in an immunocompetent mouse model for glioblastoma and could indeed demonstrate the induction of endogenous antitumor immunity following therapy with CAR-engineered NK-92 cells (69). In this model, the majority of mice carrying syngeneic intracranial GL261/ErbB2 glioblastomas were cured upon repeated intratumoral injection of ErbB2-specific NK-92/5.28.z cells, while unmodified parental NK-92 cells were unable to inhibit tumor progression (Figure 4A). Human NK-92 and CAR NK-92 cells do not permanently engraft in immunodeficient mice and are quickly rejected by immunocompetent animals (67, 69, 124, 125). Nevertheless, without any further treatment, all mice that were cured from their initial tumors also rejected a rechallenge with GL261/ErbB2 cells injected into the other brain hemisphere 4 months after initial therapy, mediated by an endogenous memory immune response induced in the animals by initial treatment with NK-92/5.28.z (69). Sera from these mice contained IgG antibodies reactive with both GL261/ErbB2 and ErbB2-negative, but otherwise isogenic GL261 cells (Figure 4B), indicating that the induced protective antitumor immune response was broadly directed against the glioblastoma cells and not limited to the CAR target antigen. Accordingly, mice that were cured of GL261/ErbB2 tumors also rejected a subsequent challenge with GL261 cells (Zhang et al., unpublished data). When animals that had rejected the initial tumor and the first rechallenge with GL261/ErbB2 were injected once again with GL261/ErbB2 cells but this time after depletion of CD4^+^ and CD8^+^ T cells, tumors formed in a large proportion of the mice. This demonstrates that protective immunity induced by initial treatment with NK-92/5.28.z cells was also dependent on T-cell memory (Figure 4C). Similarly, in a later study by Boissel et al., intratumoral injection with NK-92 cells expressing a CAR specific for murine CD19 induced protective antitumor immunity in a syngeneic A20 lymphoma model in immunocompetent mice (126).

These data suggest that the release of tumor antigens through the cytotoxic activity of CAR NK-92 cells, most likely augmented by the demonstrated CAR-induced production of high levels of pro-inflammatory cytokines (67, 69, 71, 74), can not only induce a humoral immune response directed against the tumor but also enhance cross-presentation of tumor antigens by murine DCs for the activation of tumor-specific CTLs. In a clinical setting, this may be further enhanced by IFN-γ, which is released at high levels by activated CAR NK-92. In murine models, the effects of human IFN-γ are limited due to the species-specificity of IFN-γ/IFNGR1 interactions (127, 128). Of note, apoptotic tumor cells have been shown to be superior to cell lysates or tumor cell RNA in inducing a tumor-specific T-cell response (129, 130). Hence, tumor-cell apoptosis induced by the release of cytotoxic granules from activated CAR NK-92 cells provides tumor antigens in a most effective form for uptake, processing, and presentation by DCs. Whether this vaccine or adjuvant effect of CAR NK-92 cells is a particular consequence of direct intratumoral administration as performed in our study and the study by Boissel et al. (69, 126) is subject of ongoing investigations.

## General Safety Aspects of CAR NK-92 Cells

In addition to the well-defined chimeric receptor, polyclonal CAR T cells carry endogenous, MHC-restricted T-cell receptors (TCRs) of unknown specificity. In an autologous setting, it can be expected that due to thymic selection, only very few autoreactive T cells are present in the periphery. Nevertheless, in donor-derived CAR T cells, CAR-induced activation and expansion may accidentally result in increased TCR-mediated reactivity with the recipient’s tissues, leading to severe GvHD (131). Unlike T cells, NK cells do not carry genetically rearranged clonogenic receptors. Ligands for germline-encoded activating NK-cell receptors are typically upregulated only by stressed cells after virus infection or malignant transformation, which is the basis of the NK cells’ intrinsic antitumor activity (2). Hence, the activating receptors NKp30, NKp46, and NKG2D expressed by NK-92 can be expected to contribute to the antitumor activity of CAR-engineered variants rather than causing adverse effects (34). The natural cytotoxicity of NK-92 is largely retained by CAR-expressing variants as demonstrated in different studies using K562 cells as targets (23, 49).

Donor lymphocyte infusion with allogeneic NK cells is mostly performed in the context of hematopoietic stem cell transplantation and generally considered safe, without a high risk of inducing GvHD (20). Nevertheless, depending on donor selection and *ex vivo* activation, development of acute GvHD after NK DLI has been described, attributed to NK-dependent augmentation of T-cell alloreactivity (132). In two clinical trials with NK-92 cells, only mild infusion-related side effects were noted, while no severe treatment-related toxicities were observed even at a cell dose as high as 1 × 10^10^/m^2^ body surface (31, 35, 36). While HLA-specific antibodies reactive with the allogeneic cells were found in some patients after NK-92 therapy, this was not linked to adverse effects. To prevent potential engraftment of NK-92 that was initially derived from a non-Hodgkin lymphoma patient (33), the cells were irradiated with 10 Gy prior to infusion, prohibiting further proliferation but only resulting in a gradual decline of cytotoxicity over several days (31). Since it is presently unknown whether non-irradiated NK-92 cells have the potential to form secondary lymphoma in a human host, irradiation of the cells before infusion will also be included as a safety measure in a planned phase I clinical trial investigating intratumoral injection of ErbB2-specific NK-92/5.28.z cells in patients with recurrent ErbB2-positive glioblastoma (69, 133). Like parental NK-92, CAR-engineered NK-92 variants transiently retain specific cytotoxicity after γ-irradiation with 10 Gy, with unchanged *in vitro* and *in vivo* antitumor activity (23, 56, 67, 69). This extends to the immunostimulatory activity of CAR NK-92 cells, which was also found for irradiated ErbB2-specific NK-92/5.28.z cells in an immunocompetent glioblastoma mouse model similar to the one described in Figure 4 (Zhang et al., unpublished data). Hence, in contrast to CAR T cells, which are capable of uncontrolled *in vivo* expansion, the effective dose of CAR NK-92 cells can be tightly managed to establish a therapeutic window, albeit at the price of potentially higher cell numbers and more frequent treatment intervals needed.

A major concern with CAR T-cell therapy is cytokine release syndrome (CRS) frequently observed in clinical trials with CD19-specific effector cells, which can be severe and even cause fatalities. IL-6 production and IL-6 trans-signaling after massive activation of infused CAR T cells were found to play a critical role in CRS (134). CRS can be managed with the IL-6 receptor (IL-6R) blocking antibody tocilizumab or steroid treatment (134–136). While the latter inhibits CAR T-cell expansion and activity, it constitutes an important option for patients who do not respond to the IL-6R antagonist. In contrast to CAR T cells, activated CAR NK-92 cells do not produce measurable amounts of IL-6 and IL-4 as demonstrated for NK-92 variants targeted to EGFR, EGFRvIII, ErbB2, or CD19 (67, 69, 71, 74). Instead, upon CAR activation, these cells secrete high levels of IFN-γ, macrophage inflammatory protein (MIP)-1α (CCL3), GM-CSF, and moderate levels of TNF-α. This cytokine/chemokine profile appears more favorable and less likely to induce CRS, while supporting the CAR NK-92-induced activation of endogenous antitumor immunity described above.

## Potential On-Target/Off-Tumor Toxicity

Chimeric antigen receptor effector cells specifically targeting mutated tumor antigens and viral antigens not expressed in normal tissues do not carry the risk of inducing on-target/off-tumor toxicity. This safety feature is given for CAR NK-92 cells selective for the tumor-specific EGFR mutant EGFRvIII frequently expressed in glioblastoma (71) and genetically modified NK-92 cells recognizing an epitope of the EBV latent protein EBNA3C in complex with HLA-A2 (78). However, most CARs currently available are directed to non-mutated self-antigens differentially expressed by the cancer cells. Consequently, there is a possibility for on-target/off-tumor activity against antigen-positive healthy tissues, which can result in severe toxicities. B-cell aplasia is typically observed after CD19 CAR T-cell therapy but can easily be managed by infusion of immunoglobulins. This may be different if a tumor-associated target antigen is also present in vital tissues. In a clinical trial conducted at the National Cancer Institute, a fatal adverse event occurred after infusion of autologous T cells modified to express an ErbB2-specific third generation CAR based on trastuzumab (137). Although antigen-independent CAR activation due to the combination of three signaling domains (CD137, CD28, CD3ζ) cannot be excluded (46), massive T-cell activation and respiratory failure immediately after CAR T-cell infusion may have been triggered at least in part by ErbB2 expressed at low levels on normal lung epithelium.

In addition to CAR affinity, the location of the CAR-binding epitope within the target antigen can play a decisive role in effector cell activation and influence on-target/off-tumor effects (82, 138). In the case of CAR T cells, CARs directed to membrane-distal epitopes were shown to be superior in binding but less potent in mediating activation than CARs directed to membrane-proximal epitopes of the same antigen (82, 83). We recently showed a similar effect for CAR NK-92 cells targeting EGFR, where a second-generation CAR based on antibody cetuximab, which interacts with domain III of EGFR mediated more potent cytotoxicity than an otherwise identical CAR based on antibody R1 that recognizes an epitope within the N-terminal EGFR domain I (71). The trastuzumab-derived ErbB2-specific scFv antibody fragment used by Morgan et al. binds to the juxtamembrane region (domain IV) of the target receptor (137, 139). In contrast, antibody FRP5 used for the generation of ErbB2-specific NK-92/5.28.z cells recognizes a discontinuous epitope within domain I of ErbB2 facing away from the cell surface (44, 140). Consequently, FRP5-based CARs are less likely than trastuzumab-based CARs to get activated by ErbB2 expressed at moderate levels, which is supported by data from a clinical trial in sarcoma patients with ErbB2-specific T cells carrying an FRP5-based second-generation CAR, where no on-target/off-tumor toxicities were observed (141).

**Figure 5:**
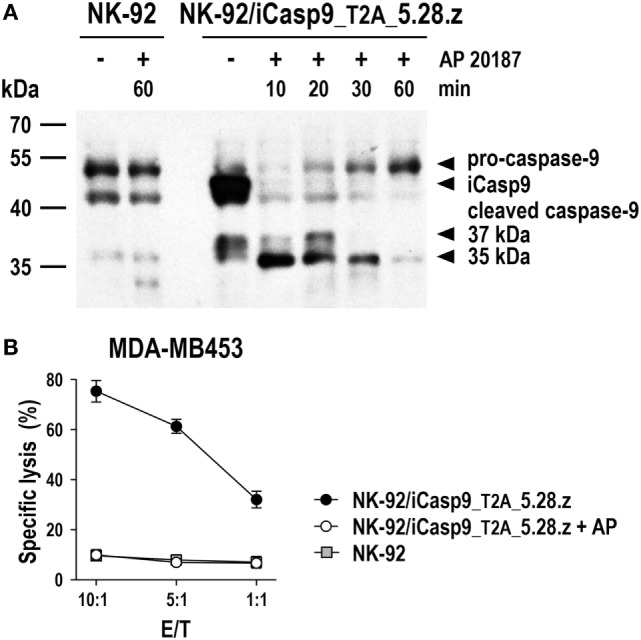
**Inducible caspase-9 (iCasp9) as a safety switch for CAR-engineered NK-92 cells**. **(A)** NK-92 cells transduced with a lentiviral vector that encodes iCasp9 and ErbB2-specific CAR 5.28.z separated by a *Thosea asigna* virus self-cleaving peptide (T2A) (NK-92/iCasp9_T2A_5.28.z) were incubated in the presence of 10 nM of the homodimerizer AP20187 for iCasp9 activation. Lysates of cells collected after 10, 20, 30, or 60 min of exposure to AP20187 were subjected to SDS-PAGE and subsequent immunoblotting with a caspase-9-specific antibody. Lysates of NK-92/iCasp9_T2A_5.28.z cells kept without dimerizer and parental NK-92 cells incubated in the absence or presence of AP20187 served as controls. **(B)** Cytotoxicity of NK-92/iCasp9_T2A_5.28.z cells against ErbB2-overexpressing MDA-MB453 breast carcinoma cells was investigated in flow cytometry-based cytotoxicity assays after co-incubation of NK cells and tumor cells at different effector to target ratios (E/T) for 2 h in the absence (filled circles) or presence of AP20187 (open circles). Parental NK-92 cells were included for comparison (gray boxes). Mean values ± SEM are shown; *n* = 2.

Irradiated CAR NK-92 cells do not expand and persist *in vivo*. Hence, they may even be applicable in a clinical setting to target more abundantly expressed self-antigens such as non-mutated EGFR (70–72), since side effects due to reactivity with normal tissues would be expected to be transient. In the future, more sophisticated safety measures like expression of inducible caspase-9 (iCasp9) as a suicide gene may thereby replace γ-irradiation of NK-92 and CAR NK-92 cells, allowing to rapidly eliminate the cells in case of toxicities, but also to extend *in vivo* activity with a reduction in the cell dose and treatment frequency needed. iCasp9 represents a fusion of the human FK506-binding protein FKBP12 harboring an F36V mutation, and truncated human caspase-9 lacking the caspase activation and recruitment domain (142). In the presence of otherwise inert FK506 analogs such as AP1903 or AP20187, iCasp9 dimerizes, inducing caspase activation and apoptotic cell death. NK-92 cells coexpressing iCasp9 and a CAR are viable in the absence of dimerizer and retain high and specific CAR-mediated cytotoxicity. In contrast, addition of AP20187 rapidly induces activation of iCasp9 and cleavage of endogenous caspases, precluding any further cell killing by the CAR-engineered cells (Figure 5).

## Conclusion and Future Perspectives

Over the past 25 years, NK-92 cells have transformed from a readily available model for studies on human NK cell biology to a promising cell therapeutic for applications in adoptive cancer immunotherapy. As outlined above, genetic modification of NK-92 with CARs has emerged as a successful strategy to enhance the cells’ intrinsic antitumor activity and provide them with the capacity for selective target recognition. The ability of CAR NK-92 cells to bypass the immunosuppressive effects of TGF-β and hypoxia in preclinical studies and to enhance or initiate adaptive antitumor immunity is encouraging (69). Nevertheless, the potential impact of immunosuppressive factors like IDO, PGE2, IL-4, NO, and ROS abundant in the tumor microenvironment has not yet been investigated. Concurrent interference with these mechanisms may offer an opportunity to further improve the direct antitumor activity of CAR NK-92 and enhance their immunostimulatory potential. Also, clarifying the relevance of checkpoint regulators such as PD-1 for CAR NK-92 functionality (74) and investigating combination therapies with checkpoint inhibitors and other immunomodulatory regimens appears warranted. Specific cytotoxicity of the NK cells may be enhanced by ectopic expression of components of the cytolytic machinery (143). Chemoattractants like CXCR3 ligands and chemerin can increase accumulation of NK cells at tumor sites (144, 145) and modulation of chemokine receptor expression would likely augment the tumor homing capability of CAR NK-92 cells (146).

Clinical responses seen in individual patients treated with parental NK-92 and ease of improvement by genetic modification with Fc receptors and CARs not only increased efforts of academic researchers to design tailor-made variants for specific disease entities and target antigens but also sparked commercial interest, which is essential to address the challenges associated with standardization of such cell products, large-scale expansion, logistics for distribution, and advanced clinical development (32, 90). Current efforts in the CAR T-cell field are aimed at generating similar universal cell products by eliminating endogenous TCR and MHC with the help of sophisticated gene editing procedures (147–149). This underscores the relevance of truly off-the-shelf CAR cell products like CAR NK-92 for broader applicability of this therapeutic strategy. Early phase clinical trials with CAR NK-92 cells are expected to commence in the near future. Insights from these studies will be essential to judge the therapeutic potential of CAR NK-92 in comparison to *ex vivo* expanded and CAR-engineered primary NK cells and determine the direction of further development.

## Ethics Statement

Animal experiments were approved by the responsible government committee (Regierungspräsidium Darmstadt, Darmstadt, Germany) and were conducted according to the applicable guidelines and regulations.

## Author Contributions

All authors made substantial contributions to the conception and design of this review article and critically evaluated the cited literature. CZ and SO performed experiments and analyzed data. CZ, PO, AW, AL, and WW drafted the initial version of the manuscript with support from all other co-authors. All authors revised the manuscript and approved the final version of this study. All authors agree to be accountable for the content of this work.

## Conflict of Interest Statement

CZ, TT, and WW are named as inventors on patents and patent applications in the field of cancer immunotherapy owned by their respective institutions. The authors declare that own research described in this review article was conducted in the absence of any other commercial or financial relationships that could be construed as a potential conflict of interest. The reviewer AC declared a shared affiliation, though no other collaboration, with several of the authors CZ, TT and WW to the handling Editor, who ensured that the process nevertheless met the standards of a fair and objective review.
